# Association of *HFE* genotypes with hemochromatosis-related phenotypes in the All of Us research program

**DOI:** 10.1016/j.gimo.2024.101959

**Published:** 2025-01-07

**Authors:** Nandana D. Rao, Ramal Moonesinghe, Lu Shi, Paul C. Adams, Gail P. Jarvik, Kris V. Kowdley, Laura A. Schieve, Scott D. Grosse, W. David Dotson, Muin J. Khoury

**Affiliations:** 1 Division of Blood Disorders and Public Health Genomics, National Center on Birth Defects and Developmental Disabilities, Centers for Disease Control and Prevention, Atlanta, GA; 2 Department of Medicine, Schulich School of Medicine and Dentistry, Western University, London, ON, Canada; 3 Departments of Medicine (Medical Genetics) and Genome Sciences, University of Washington Medical Center, Seattle, WA; 4 Department of Hepatology, Liver Institute Northwest, Seattle, WA; 5 National Center on Birth Defects and Developmental Disabilities, Centers for Disease Control and Prevention, Atlanta, GA

**Keywords:** C282Y homozygosity, Genetic testing, Hereditary hemochromatosis, Iron overload, Liver disease

## Abstract

**Purpose:**

Type 1 hereditary hemochromatosis (HH) can result in iron overload and liver disease if not detected and treated early. Most cases are found among people homozygous for *HFE* p.Cys282Tyr variants. Compound heterozygosity with the *HFE* p.His63Asp variant is associated with disease to a lesser degree. We sought to examine the association of *HFE* variation with HH-related phenotypes and assess the prevalence of testing and diagnosis of HH using All of Us data.

**Methods:**

We used data from 133,978 participants with genetic information linked to medical records. For different *HFE* genotypes, we examined the prevalence of HH diagnosis codes and related biochemical and clinical phenotypes.

**Results:**

Among participants who were p.Cys282Tyr homozygotes, the prevalence of HH diagnosis codes was 22.6% among males and 15.6% among females. Serum transferrin-iron saturation measures were available only for 31.4% of males and 21.1% of females who were p.Cys282Tyr homozygotes. Liver disease, including cirrhosis or hepatocellular carcinoma, was present more among males who were p.Cys282Tyr homozygotes compared with males with no p.Cys282Tyr or p.His63Asp variants (15.5% vs 8.5%, *P* = .0001). Of the 71 participants who were p.Cys282Tyr homozygotes with indication of liver disease, 32 (45.1%) did not have a serum transferrin-iron saturation measure, and 37 (52.1%) did not have diagnosis codes for HH.

**Conclusion:**

Limited serum transferrin-iron saturation measures or HH diagnosis codes among p.Cys282Tyr homozygotes, even those with liver disease, suggests potential undertesting and underdiagnosis of type 1 HH in clinical practice and a need for improved awareness, education, and testing around HH.

## Introduction

Type 1 hereditary hemochromatosis (HH) is an autosomal recessive genetic disorder with incomplete penetrance associated with variation in the *HFE* (homeostatic iron regulator, HGNC:4886, GRCh38 reference) gene and characterized by iron overload due to excessive absorption of dietary iron.^1^, ^2^, ^3^ Iron overload can lead to increased iron deposition in organs, resulting in cirrhosis, liver cancer, and increased mortality.^4^ Most cases are among individuals homozygous for the *HFE* c.845G>A substitution (GRCh38.p14, NC_000006.12:g.26092913G>A, p.Cys282Tyr, rs1800562).^5^^,^^6^ Compound heterozygosity with the *HFE* c.187C>G substitution (GRCh38.p14, NC_000006.12:g.26090951C>G, p.His63Asp, rs1799945) is associated with lower penetrance of disease, and other rare pathogenic variants have also been described.^1^^,^^5^, ^6^, ^7^, ^8^, ^9^, ^10^, ^11^, ^12^, ^13^ The frequency of *HFE* p.Cys282Tyr and p.His63Asp variants differs by ancestry, and both are increased in those of European genetic ancestry.^14^

In contemporary clinical practice, diagnosis of HH is based on a positive genetic test (p.Cys282Tyr homozygosity) in the setting of associated signs and symptoms of iron overload.^1^^,^^15^ However, HH is sometimes diagnosed without a genetic test based on biochemical measures of iron alone.^15^ An algorithm proposed by the American College of Gastroenterology suggests diagnosis of HH first through testing for elevated serum transferrin-iron saturation (TS) or serum ferritin (SF) followed by *HFE* testing.^2^

In the UK Biobank, a large longitudinal study of volunteers registered with the UK National Health Service who were aged 40 to 70 years at registration, males who were p.Cys282Tyr homozygotes had a higher modeled cumulative incidence of liver disease to age 80 years compared with males with no p.Cys282Tyr or p.His63Asp variants (20.3% vs 8.3%), as well as more joint replacements (27.9% vs 17.1%).^4^ Excess liver disease was also found among females who were p.Cys282Tyr homozygotes compared with females with no p.Cys282Tyr or p.His63Asp variants (8.9% vs 6.8%).^4^ Homozygosity for p.Cys282Tyr has previously been reported to be associated with delirium, dementia, and Parkinson's disease, as well as a slightly increased incidence of prostate cancer.^4^^,^^16^

Early diagnosis and phlebotomy treatment that safely removes excess iron from the body may result in fewer harmful impacts of HH. Phlebotomy has been observed to reverse hepatic fibrosis in most asymptomatic individuals without cirrhosis who were p.Cys282Tyr homozygotes.^17^^,^^18^

Although gene disease associations seen in the UK Biobank are likely to be reflected in a US population, differences in health systems and practices across countries may affect how HH is diagnosed. The All of Us research program (AoU) provides an opportunity to further examine cross-sectional associations of *HFE* variation with HH-related phenotypes in a US population and assess the prevalence of testing and diagnosis of HH.

## Materials and Methods

The AoU aims to improve biomedical research and health outcomes by collecting health questionnaires, electronic health records (EHR), physical measurements, digital health measures, and biospecimen data from a diverse group of people in the United States.^19^ Participants provide written informed consent for sharing their data. To protect participant privacy, the AoU Data and Statistics Dissemination Policy states that data or aggregate statistics corresponding to fewer than 20 participants cannot be published, nor can data or statistics be reported that allow a participant count of less than 20 to be derived.^20^

We used the AoU Controlled Tier Dataset V7, which includes participants who enrolled and consented to the research program from May 2018 to July 1, 2022. Our analysis included participants with nonmissing *HFE* p.Cys282Tyr and p.His63Asp genetic data and sex at birth information (male or female).

Sex at birth, race, and ethnicity were obtained using self-reported information generated for the AoU Controlled Tier Dataset. Age was calculated by subtracting the participant’s year of birth from the year of analysis (2023).

Genetic information in the AoU Controlled Tier Dataset includes microarray genotyping (examines specific sites of the genome) and genome sequencing (examines the entirety of the genome) data. We included participants with data from either method in our analysis. However, if microarray and short-read results were available for the same person (78.4% of participants with nonmissing *HFE* data), short-read results were used to avoid duplication. For *HFE* p.Cys282Tyr data, array and short-read results matched in 99.9% of instances. We grouped participants according to the following genotypes: p.Cys282Tyr homozygotes, p.Cys282Tyr/p.His63Asp compound heterozygotes, p.Cys282Tyr simple heterozygotes, p.His63Asp homozygotes, p.His63Asp simple heterozygotes, and no p.Cys282Tyr or p.His63Asp variants. For our purposes, compound heterozygous indicates that the individual carried both the p.Cys282Tyr and p.His63Asp variants. However, we were unable to determine the phase of these variants.

We sought to make the no p.Cys282Tyr or p.His63Asp variants group comparable to the variant positive groups by restricting our analysis to participants who self-reported as non-Hispanic White. This restriction removed 48 people in the p.Cys282Tyr homozygote group across males and females.

We queried linked EHR data for biochemical and clinical phenotypes related to HH and HH diagnosis codes (Supplemental Table 1 lists codes used in queries). Biochemical measures of interest included TS and SF. If multiple biochemical measures were available for one person, the most recent measure was used. High TS was defined as greater than 50% for males and 45% for females.^1^ High SF was defined as greater than 300 μg/L for males and 200 μg/L for females).^1^

Clinical phenotypes of interest included brain-related conditions (dementia, delirium, and Parkinson's disease), cardiac conditions, chronic fatigue, depression, diabetes, liver conditions, prostate cancer, osteoarthritis, osteoporosis, and rheumatoid arthritis. Phenotypes were chosen because of associations with p.Cys282Tyr homozygosity reported in the UK Biobank and importance to patients with HH.^4^^,^^21^, ^22^, ^23^ For clinical phenotypes and HH diagnosis codes, we looked for at least 2 records on different days reporting the phenotype for an individual.

We also examined survey data for self-reported diagnoses of phenotypes of interest (Supplemental Table 1 lists relevant survey questions). We combined survey and EHR information to improve the sample size.

We focused our statistical analysis on participants who were p.Cys282Tyr homozygotes given the higher penetrance of p.Cys282Tyr homozygosity for HH, the American College of Medical Genetics recommendation to return this genotype when found incidentally on a genomic test, and recent proposals about genetic screening for p.Cys282Tyr homozygosity.^4^^,^^24^^,^^25^ For each biochemical and clinical phenotype of interest, we compared the p.Cys282Tyr homozygote group with the no p.Cys282Tyr or p.His63Asp variant group using a χ^2^ test with 1° of freedom and a 0.05 significance level to determine if the particular phenotype was present more often in the homozygote group. As a secondary exploratory analysis, we used χ^2^ tests with 1° of freedom to compare the presence of each biochemical or clinical phenotype in the p.Cys282Tyr/p.His63Asp compound heterozygote group with the no p.Cys282Tyr or p.His63Asp variant group.

## Results

Out of the 409,420 AoU participants with research data (total participant count as of February 2023), 249,815 (61.0%) participants had available sex at birth data, genetic data for *HFE* variants p.Cys282Tyr and p.His63Asp, and linked EHR data. Overall, 133,978 (53.6%) participants were non-Hispanic White (Table 1), and 96,796 (38.7%) were male. The average ages of male and female participants at the time of data abstraction were 58.4 and 54.6 years, respectively. Among AoU participants, the p.Cys282Tyr allele frequency was 4%, and the p.His63Asp allele frequency was 11%. Among self-reported non-Hispanic White participants only, p.Cys282Tyr allele frequency was 6.1%, and p.His63Asp frequency was 15%. The frequency of p.Cys282Tyr homozygosity was 0.4% across non-Hispanic White males and females.

**Table 1:** Demographics of participants in the All of Us research program

Demographics/Genotype	Male							Female						
	p.Cys282Tyr/p.Cys282Tyr	p.Cys282Tyr/p.His63Asp	p.Cys282Tyr/--	p.His63Asp/p.His63Asp	p.His63Asp/--	No p.Cys282Tyr or p.His63Asp Variants	Total	p.Cys282Tyr/p.Cys282Tyr	p.Cys282Tyr/p.His63Asp	p.Cys282Tyr/--	p.His63Asp/p.His63Asp	p.His63Asp/--	No p.Cys282Tyr or p.His63Asp Variants	Total
N^a^	257	1108	6299	1502	17459	70171	96796	376	1677	9871	2336	27931	110828	153019
Non Hispanic white	239	970	5110	1175	12354	33493	5334 1	346	1437	7734	1737	18844	50539	80637
Age at abstraction^b^	62.9 (15.7)	62.0 (16.7)	60.5 (16.8)	60.3 (17.1)	59.5 (17.1)	57.8 (16.6)	58.4 (16.8)	59.6 (15.6)	57.0 (16.7)	56.5 (17.1)	56.4 (17.4)	55.3 (17.2)	54.2 (16.8)	54.6 (16.9)

a Participants included people with available *HFE* genetic data, male or female sex at birth, and linked EHR data.

b Mean (SD).

Participants who were p.Cys282Tyr homozygotes were more likely to have HH diagnosis codes compared with participants with no p.Cys282Tyr or p.His63Asp variants. Among males and females who were p.Cys282Tyr homozygotes, 22.6% and 15.6%, respectively, had HH diagnosis codes (Table 2). The average age of HH diagnosis was 59.3 years (standard deviation 12.2) among male p.Cys282Tyr homozygotes and 57.6 years (standard deviation 13.4) among female p.Cys282Tyr homozygotes. A lower percentage of participants who were p.Cys282Tyr/p.His63Asp compound heterozygous had HH diagnosis codes present (3% in males with average age of diagnosis at 58.5 years and a standard deviation of 12.2, 1.7% in females with average age of diagnosis at 52.5 years and a standard deviation of 12.3).

**Table 2:** Comparison of lab measures and disease conditions based on *HFE* genotype [N (%)]

Biochemical and Clinical Measures/Genotype	Male							Female						
	p.Cys282Tyr/p.Cys282Tyr	p.Cys282Tyr/p.His63Asp	p.Cys282Tyr/--	p.His63Asp/p.His63Asp	p.His63Asp/--	No p.Cys282Tyr or p.His63Asp variants	P-value^a^	p.Cys282Tyr/p.Cys282Tyr	p.Cys282Tyr/p.His63Asp	p.Cys282Tyr/--	p.His63Asp/p.His63Asp	p.His63Asp/--	No p.Cys282Tyr or p.His63Asp variants	*P* value^a^
N	239	970	5110	1175	12354	33493	--	346	1437	7734	1737	18844	50539	--
*Biochemical*														
N with SF	85 (35.6)	233 (24.0)	1093 (21.4)	262 (22.3)	2504 (20.3)	6994 (20.9)	<0.0001 0.018	92 (26.6)	341 (23.7)	1688 (21.8)	406 (23.4)	4360 (23.1)	11688 (23.1)	0.128 0.593
N with high SF among those with SF test	<20 (<23.5)	52 (22.3)	192 (17.6)	53 (20.2)	435 (17.4)	1188 (17.0)	0.900 0.034	<20 (<21.7)	56 (16.4)	190 (11.3)	58 (14.3)	534 (12.3)	1334 (11.4)	0.415 0.004
N with TS	75 (31.4)	181 (18.7)	900 (17.6)	211 (18.0)	2049 (16.6)	5760 (17.2)	<0.0001 0.235	73 (21.1)	290 (20.2)	1368 (17.7)	352 (20.3)	3536 (18.8)	9498 (18.8)	0.274 0.185
N with high TS among those with TS test	42 (56.0)	43 (23.8)	60 (6.7)	27 (12.8)	111 (5.4)	202 (3.5)	<0.0001 <0.0001	42 (57.5)	55 (19.0)	101 (7.4)	43 (12.2)	204 (5.8)	354 (3.7)	<0.0001 <0.0001
*Clinical*														
Hereditary hemochromatosis^b^	54 (22.6)	29 (3.0)	<20 (<0.4)	<20 (<1.7)	<20 (<0.2)	<20 (<0.06)	<0.0001 <0.0001	54 (15.6)	25 (1.7)	<20 (<0.3)	<20 (<1.2)	<20 (<0.1)	<20 (<0.04)	<0.0001 <0.0001
Liver condition^c^	37 (15.5)	93 (9.6)	484 (9.5)	104 (8.9)	1030 (8.3)	2855 (8.5)	0.0001 0.243	34 (9.8)	132 (9.2)	617 (8.0)	162 (9.3)	1543 (8.2)	4020 (8.0)	0.199 0.090

a First *P* value listed compares participants who are p.Cys282Tyr homozygotes with participants with no p.Cys282Tyr or p.His63Asp variants. Second *P* value listed compares participants who are p.Cys282Tyr and p.His63Asp compound heterozygotes with participants with no p.Cys282Tyr or p.His63Asp variants.

b Based on International Classification of Diseases codes in the electronic health record.

c Based on International Classification of Diseases codes in the electronic health record and responses to the personal and family health history survey.

Among self-reported non-Hispanic White participants who were p.Cys282Tyr homozygotes, 31.4% of males and 21.1% of females had a TS measure (Table 2). Although a greater percentage of males who were p.Cys282Tyr homozygotes had a TS measure compared with males with no p.Cys282Tyr or p.His63Asp variants (31.4% vs 17.2%, *P* < .0001), the same was not true for females (21.1% vs 18.8%, *P* = .27). A comparison of TS between genotype groups showed that among males, a greater percentage of participants were p.Cys282Tyr homozygotes with high TS compared with participants with no p.Cys282Tyr or p.His63Asp variants (56% vs 3.5%, *P* < .0001). Similarly, among females, there was a greater percentage of participants who were p.Cys282Tyr homozygotes with high TS compared with participants with no p.Cys282Tyr or p.His63Asp variants (57.5% vs 3.7%, *P* < .0001). Of the participants who were p.Cys282Tyr homozygotes with high TS, 24 of 42 (57.1%) males and 23 of 42 (54.8%) females had HH diagnosis codes.

Males who were p.Cys282Tyr homozygotes also had a higher percentage of liver conditions compared with males with no p.Cys282Tyr or p.His63Asp variants (15.5% vs 8.5%, *P* = .0001; Table 2). Roughly a fourth of males and females who were p.Cys282Tyr homozygotes with reported liver disease had diagnosis codes for either hepatocellular carcinoma or cirrhosis compared with less than a fifth of participants with reported liver disease and no p.Cys282Tyr or p.His63Asp variants (exact numbers not provided due to small sample in homozygote group, *P* = .031). Additionally, of the 71 participants who were p.Cys282Tyr homozygotes with an indication of liver disease, 32 (45.1%) had no recorded TS measure and 37 (52.1%) did not have HH diagnosis codes. Results for additional phenotypes are listed in Supplemental Table 2.

## Discussion

As expected, participants who were p.Cys282Tyr homozygotes were more likely to have high TS or liver disease compared with participants with no p.Cys282Tyr or p.His63Asp variants. HH diagnosis codes were also reported most often among people who were p.Cys282Tyr homozygotes. The presence of HH codes among some individuals with no p.Cys282Tyr variants could reflect HH due to rare variants in *HFE* or other genes. However, it could also be indicative of misdiagnosis or misclassification, suggesting that a more stringent algorithm, perhaps incorporating International Classification of Diseases codes, as well as lab measures or genetic testing, may be needed to determine true HH diagnoses in the EHR.

The frequency of the p.Cys282Tyr allele was 6.1% in AoU compared with 7.3% in the UK Biobank.^16^ The prevalence of HH diagnosis codes among participants who were p.Cys282Tyr homozygotes in this study (22.6% among males, 15.6% among females) is also similar to previous reports. After an average of 7-year follow-up of UK Biobank participants, HH had been diagnosed in 21.7% of males and 9.8% of females who were p.Cys282Tyr homozygotes.^22^ Data from the Electronic Medical Records and Genomics (eMERGE) Network, a consortium of adult and pediatric cohorts in the United States with DNA repositories linked to electronic medical records, showed HH diagnoses in 24.4% and 14.0% of males and females, respectively, who were p.Cys282Tyr homozygotes and of European descent.^26^ After an average of 7-year follow-up of UK Biobank participants, HH had been diagnosed in 21.7% of males and 9.8% of females who were p.Cys282Tyr homozygotes.^22^

High TS measures among participants who were p.Cys282Tyr homozygotes in this study also support prior research. An eMERGE analysis reported high TS among 10 of 10 (100%) male participants and 4 of 8 (50%) female participants who were p.Cys282Tyr homozygotes.^26^ In the AoU, high TS was seen in 42 of 75 (56%) males and 42 of 73 (57.5%) females who were p.Cys282Tyr homozygotes. However, TS measures were available for only 31.4% and 21.1% of males and females, respectively, who were p.Cys282Tyr homozygotes, and of these participants with high TS, just over half had HH diagnosis codes. This suggests undertesting for iron overload and serum iron markers among people who are p.Cys282Tyr homozygotes, likely resulting in missed HH diagnoses. Potential HH underdiagnosis has also been reported by Geisinger’s MyCode, a study of biobank data from a central Pennsylvania health care system.^24^ *HFE* genetic screening of MyCode participants identified 201 people who were p.Cys282Tyr homozygotes, 144 (71.6%) of whom were not aware of their genotype and 53 of 144 (36.8%) of whom had evidence of iron overload.^24^

High SF was not seen more among participants who were p.Cys282Tyr homozygotes compared with participants with no p.Cys282Tyr or p.His63Asp variants (males: <23.5% vs 17%, females: <21.7% vs 11.4%). However, high SF is not unique to iron overload but can be present because of chronic inflammation, alcohol consumption, or metabolic syndromes, conditions that may have been present in the no p.Cys282Tyr or p.His63Asp variants groups.^1^^,^^27^

Similar to our study, a higher incidence of liver disease was seen among males who were p.Cys282Tyr homozygotes compared with males with no p.Cys282Tyr or p.His63Asp variants (8.1% vs 3.3%) over an average 13.3-year follow-up in the UK Biobank.^4^ Among AoU participants, liver disease was prevalent in 15.5% of males who were p.Cys282Tyr homozygotes compared with 8.5% with no p.Cys282Tyr or p.His63Asp variants. Although the comparison between an incident and a prevalent disease is challenging, results consistently show more frequent liver complications among individuals who are p.Cys282Tyr homozygotes. Of note, in the AoU, liver disease was present in participants who were p.Cys282Tyr homozygotes with no recorded TS measures or HH diagnosis codes, potentially indicating unrecognized and untreated HH. Consistent with previous UK Biobank work, liver disease was not as present in female p.Cys282Tyr homozygotes compared with male p.Cys282Tyr homozygotes.^4^ This difference is thought to be due to iron loss occurring in females during menstruation.^28^, ^29^, ^30^

Our study was limited by the cross-sectional nature of the AoU data set, which prevented analyzing incident diagnoses. Limited sample size restricted our ability to investigate differences in biochemical and clinical phenotypes across *HFE* genotypes or in individuals who did not self-report as non-Hispanic White or by genetic ancestry. Participants with HH symptoms may have been more likely to have testing completed for HH and HH diagnosis codes. Age is also an important factor in HH, and it is possible that more people will present with HH as they age. Limited sample size prevented the presentation of data stratified by age groups. Additionally, available EHR information may be incomplete if participants received care at multiple sites, and the presence of diagnosis codes in the EHR may not necessarily indicate the clinical diagnosis of a condition.

Results from the AoU support previous findings regarding the increased burden of excess iron and liver disease among people who are p.Cys282Tyr homozygotes and suggest undertesting and underdiagnosis of HH.^4^^,^^24^^,^^26^ If HH is diagnosed early, high iron levels can be addressed and liver disease prevented. Further work to educate providers about HH signs, symptoms, and diagnosis, improved efforts to increase genetic testing of adult first-degree biological relatives of people known to be p.Cys282Tyr homozygotes, and pilot programs examining p.Cys282Tyr genetic screening may assist with reducing morbidity and mortality from HH.

## Data Availability

Access to the All of Us Researcher Workbench Controlled Tier data set is free and requires the creation of an account through an institution with a Data Use and Registration Agreement, completion of ethics training, and signed agreement to a data user code of conduct.

## Conflict of Interest

Kris V. Kowdley receives research support from 10.13039/100020154Protagonist Therapeutics. All other authors declare no conflicts of interest.
