# Supplementary material for: Association of HFE genotypes with hemochromatosis-related phenotypes in the All of Us research program
Source: Genet Med Open. 2025 Jan 7;3:101959. doi: 10.1016/j.gimo.2024.101959 (PMC11875803; doi:10.1016/j.gimo.2024.101959)
Supplement: Supplemental Tables [file mmc1.pdf]

Supplementary Material

Supplementary Table 1: Diagnosis and procedure codes used to extract biochemical and clinical phenotypes from the EHR

Biochemical and Clinical Phenotypes	Data Type	Code(s)
Transferrin saturation	LOINC	2502-3; 14801-5
	SNOMED	165730006
Serum ferritin	LOINC	24373-3; 2276-4; 20567-4; LP15568-6; LP385080-9; LP385083-3
	SNOMED	489004; 275759006
Atrial fibrillation or arrythmia	ICD-9-CM	427.3; 427.4
	ICD-10-CM	I48; I49
	Survey	Including yourself, who in your family has had atrial fibrillation (or a-fib) or atrial flutter (or a-flutter)?
Brain outcome	ICD-9-CM	290.4; 293.0; 294.1; 294.2; 331.0; 332
	ICD-10-CM	F00; F01; F02; F02.3; F03; F05; G20; G30
	Survey	Including yourself, who in your family has had dementia (includes Alzheimer's, vascular, etc.)?<br>Including yourself, who in your family has had Parkinson's disease?
Cardiomyopathy	ICD-9-CM	425[a]
	ICD-10-CM	I42
Chronic fatigue	ICD-9-CM	780.71
	ICD-10-CM	R53.82
	Survey	Including yourself, who in your family has had chronic fatigue?
Coronary heart disease (includes MI)	ICD-9-CM	410; 411; 412; 413; 414
	ICD-10-CM	I20; I21; I22; I23; I24; I25
	Survey	Including yourself, who in your family has had coronary artery/coronary heart disease?<br>Including yourself, who in your family has had a heart attack?
Depression	ICD-9-CM	296.2; 296.3; 300.4
	ICD-10-CM	F32; F33; F34.1
	Survey	Including yourself, who in your family has had depression?

Diabetes (Type 1 or 2)	ICD-9-CM	250.01; 250.11; 250.21; 250.91; 250.00; 250.10; 250.20; 250.90
	ICD-10-CM	E10; E11
	Survey	Including yourself, who in your family has had type 1 diabetes?<br>Including yourself, who in your family has had type 2 diabetes?
Heart failure	ICD-9-CM	428; 518.4
	ICD-10-CM	I50; J81
	Survey	Including yourself, who in your family has had congestive heart failure?
Hereditary hemochromatosis	ICD-9-CM	275.01
	ICD-10-CM	E83.110
Liver disease (any)	ICD-9-CM	155; 570[a]; 571[a]; 572[a]; 573[a]
	ICD-10-CM	C22; K70; K71; K72; K73; K74; K75; K76; K77;
	Survey	Including yourself, who in your family has had a liver condition (e.g., cirrhosis)?
Osteoarthritis	ICD-9-CM	715[a]
	ICD-10-CM	M15.0; M15.1; M15.2; M15.9; M16.0; M16.1; M17.0; M17.1; M18.0; M18.1; M19.0
	Survey	Including yourself, who in your family has had osteoarthritis?
Osteoporosis	ICD-9-CM	733
	ICD-10-CM	M80; M81; M811; M812; M813; M814; M815; M816; M818; M819
	Survey	Including yourself, who in your family has had osteoporosis?
Prostate cancer	ICD-9-CM	185
	ICD-10-CM	C61
	Survey	Including yourself, who in your family has had prostate cancer?
Rheumatoid arthritis	ICD-9-CM	714.0[a]
	ICD-10-CM	M05[a]; M06[a]
	Survey	Including yourself, who in your family has had rheumatoid arthritis (RA)?

a: all codes below that which is specified;

LOINC: Logical Observation Identifiers Names and Codes; SNOMED: Systematized Nomenclature of Medicine Clinical Terms; ICD-9-CM: International Classification of Diseases, Ninth Revision, Clinical Modification; ICD-10-CM: International Classification of Diseases, Tenth Revision, Clinical Modification

Supplementary Table 2: Comparison of additional disease conditions based on *HFE* genotype [N (%)]

	Male							Female						
	**p.Cys282Tyr / p.Cys282Tyr**	**p.Cys282Tyr / p.His63Asp**	**p.Cys282Tyr / --**	**p.His63Asp / p.His63Asp**	**p.His63Asp / --**	**No p.Cys282Tyr or p.His63Asp Variants**	**P-Value [c]**	**p.Cys282Tyr / p.Cys282Tyr**	**p.Cys282Tyr / p.His63Asp**	**p.Cys282Tyr / --**	**p.His63Asp / p.His63Asp**	**p.His63Asp / --**	**No p.Cys282Tyr or p.His63Asp Variants**	**P-Value [c]**
**Atrial fibrillation or arrythmia[a]**	43 (18.0)	169 (17.4)	849 (16.6)	177 (15.1)	2035 (16.5)	5414 (16.2)	0.445 0.295	27 (7.8)	160 (11.1)	745 (9.6)	176 (10.1)	1751 (9.3)	4739 (9.4)	0.317 0.025
**Brain outcome[a]**	<20 (<8.4)	<20 (<2.1)	145 (2.8)	23 (2.0)	286 (2.3)	793 (2.4)	0.885 0.022	<20 (<5.8)	<20 (1.4)	93 (1.2)	20 (1.2)	190 (1.0)	629 (1.2)	0.109 0.658
**Cardiomyopathy[b]**	<20 (<8.4)	42 (4.3)	171 (3.3)	40 (3.4)	456 (3.7)	1187 (3.5)	0.870 0.193	<20 (<5.8)	33 (2.3)	134 (1.7)	29 (1.7)	323 (1.7)	817 (1.6)	0.126 0.045
**Chronic fatigue[a]**	<20 (<8.4)	25 (2.6)	146 (2.9)	35 (3.0)	331 (2.7)	859 (2.6)	0.958 0.981	<20 (<5.8)	91 (6.3)	451 (5.8)	118 (6.8)	1176 (6.2)	3057 (6.0)	0.665 0.657
**Coronary heart disease (includes MI) [a]**	39 (16.3)	204 (21.0)	994 (19.5)	227 (19.3)	2501 (20.2)	6680 (19.9)	0.162 0.404	25 (7.2)	141 (9.8)	651 (8.4)	159 (9.2)	1548 (8.2)	4165 (8.2)	0.493 0.033
**Depression[a]**	51 (21.3)	223 (23.0)	1205 (23.6)	257 (21.9)	2821 (22.8)	7625 (22.8)	0.600 0.870	121 (35.0)	525 (36.5)	2699 (34.9)	625 (36.0)	6649 (35.3)	17750 (35.1)	0.954 0.269
**Diabetes[a]**	48 (20.1)	172 (17.7)	923 (18.1)	220 (18.7)	2270 (18.4)	6166 (18.4)	0.506 0.591	38 (11.0)	205 (14.3)	968 (12.5)	227 (13.1)	2308 (12.2)	6034 (11.9)	0.584 0.007
**Heart failure[a]**	23 (9.6)	83 (8.6)	407 (8.0)	96 (8.2)	975 (7.9)	2580 (7.7)	0.268 0.326	<20 (<5.8)	81 (5.6)	338 (4.4)	71 (4.1)	805 (4.3)	2159 (4.3)	0.316 0.012
**Osteoarthritis[a]**	71 (29.7)	228 (23.5)	1150 (22.5)	261 (22.2)	2795 (22.6)	7667 (22.9)	0.013 0.654	118 (34.1)	443 (30.8)	2259 (29.2)	539 (31.0)	5372 (28.5)	14586 (28.9)	0.032 0.105
**Osteoporosis[a]**	<20 (<8.4)	47 (4.8)	291 (5.7)	54 (4.6)	661 (5.4)	1798 (5.4)	0.418 0.476	81 (23.4)	259 (18.0)	1511 (19.5)	363 (20.9)	3651 (19.4)	10270 (20.3)	0.155 0.033
**Prostate cancer[a]**	22 (9.2)	83 (8.6)	409 (8.0)	89 (7.6)	943 (7.6)	2568 (7.7)	0.374 0.305							
**Rheumatoid arthritis[a]**	<20 (<8.4)	32 (3.3)	170 (3.3)	50 (4.3)	395 (3.2)	1190 (3.6)	0.860 0.673	<20 (<5.8)	82 (5.7)	344 (4.4)	94 (5.4)	877 (4.7)	2510 (5.0)	0.590 0.204

a: Based on International Classification of Diseases codes in the electronic health record and responses to the personal and family health history survey; b: Based on International Classification of Diseases codes in the electronic health record; c: First p-value listed compares participants who are p.Cys282Tyr

homozygotes with participants with no p.Cys282Tyr or p.His63Asp variants. Second p-value listed compares participants who are p.Cys282Tyr and p.His63Asp compound heterozygotes with participants with no p.Cys282Tyr or p.His63Asp variants.
